# Clinicopathological Parameters Predicting Malignancy in Phyllodes Tumor of the Breast

**DOI:** 10.7759/cureus.46168

**Published:** 2023-09-28

**Authors:** Atif A Hashmi, Bakhtawar Allauddin Mallick, Khushbakht Rashid, Sumbal Zafar, Shamail Zia, Umair Arshad Malik, FNU Sapna, FNU Anjali, FNU Vishal, Muhammad Irfan

**Affiliations:** 1 Pathology, Liaquat National Hospital and Medical College, Karachi, PAK; 2 Internal Medicine, Zainab Panjwani Memorial Hospital, Karachi, PAK; 3 Emergency Medicine, Al-Rayaz Hospital, Karachi, PAK; 4 Cardiology, Prime Cardiology of Nevada, Las Vegas, USA; 5 Internal Medicine, Liaquat National Hospital and Medical College, Karachi, PAK; 6 Pathology, Jinnah Sindh Medical University, Karachi, PAK; 7 Internal Medicine, Aga Khan University, Karachi, PAK; 8 Pathology, Albert Einstein College of Medicine, New York, USA; 9 Internal Medicine, Sakhi Baba General Hospital, Sukkur, PAK; 10 Public Health, Johns Hopkins Bloomberg School of Public Health, Baltimore, USA; 11 Infectious Diseases, Rochester General Hospital, Rochester, USA; 12 Statistics, Liaquat National Hospital and Medical College, Karachi, PAK

**Keywords:** tumor size, clinicopathological correlation, fibroepithelial lesion, breast tumors, phyllodes tumour

## Abstract

Introduction

Phyllodes tumor (PT) is an uncommon fibroepithelial neoplasm of the breast. It is a biphasic tumor with stromal and epithelial components, with a tendency to recur. Because of its wide range of disease manifestations, it has been subclassified into three categories, i.e., benign, borderline, and malignant, based on several histological parameters. This study was conducted to evaluate the clinicopathological features associated with malignancy in breast PTs.

Methods

We conducted a retrospective study at the Department of Histopathology at Liaquat National Hospital, Karachi, Pakistan. A total of 146 biopsy-proven cases of PTs were enrolled in the study. Clinical data were obtained from the clinical referral forms. Specimens were obtained from either lumpectomy or simple mastectomy. The specimens obtained were received at the laboratory where after gross examination, paraffin-embedded tissue blocks were prepared, which were sectioned, stained, and studied by a senior histopathologist. Pathological features, such as mitotic count, necrosis, stromal atypia, stromal overgrowth, and heterologous elements, were observed. Based on these features, the PTs were classified into benign, borderline, and malignant tumors.

Results

The mean age of the PTs in our setup was 40.65 ± 12.17 years with a mean size of 9.40 ± 6.49 cm. Malignant PT was found to be the most prevalent in our population, accounting for 63 (43.2%) cases, followed by borderline (51, 34.9%) and benign (32, 21.9%). A significant association was found between the tumor subtype and patient age, i.e., patients diagnosed with malignant and borderline PTs were found to be of older age (mean 42.82 ± 12.94 and 42.05 ± 11.31 years, respectively) than those diagnosed with benign PTs (mean age 34.12 ± 9.75 years). Moreover, malignant PTs were associated with larger tumor size (mean 11.46 ± 6.08) compared with the other two subtypes.

Conclusion

We found a significant association among patient age, tumor size, and PT subtype. Therefore, apart from the usual histological parameters, patient age and tumor size are important parameters for predicting the behavior of breast PT and should be considered for management.

## Introduction

Phyllodes tumor (PT) of the mammary gland is a rare fibroepithelial neoplasm, accounting for approximately 0.3-0.5% of all primary breast tumors [1]. According to the Surveillance, Epidemiology, and End Results (SEER) program data analysis, the average annual incidence was estimated to be 2.1 per one million women [2]. PTs are biphasic tumors with a neoplastic mesenchymal component and benign breast epithelium [3]. Histologically, these tumors are considered to be stromal derivatives because they are likely to originate from the terminal duct-lobular unit [4]. PT encompasses a wide range of fibroepithelial diseases ranging from relatively indolent, benign neoplasms to malignant tumors that have the potential to progress rapidly and metastasize [5]. Distant metastasis is a feature exclusive to malignant PTs, with the lungs (66%) and bones (28%) being the most common sites of metastasis [6]. The World Health Organization (WHO) classified PTs into three categories, namely, benign, borderline, and malignant, on the basis of five prognostically important morphological features: stromal overgrowth, stromal atypia, tumor cellularity, mitotic count, and tumor margins [7]. The incidence of benign PTs is the highest, accounting for 35-60% of cases, whereas the incidence of malignant PTs is approximately 10-25% [4-5]. Malignant PTs usually show an aggressive clinical course, with a higher potential to locally recur (approximately 30%) and distantly metastasize [8]. PTs typically present as rapidly growing benign breast lumps in females, with a peak incidence in the fourth and fifth decades of life, with an average size of 4-5 cm, with malignant PTs having the potential to grow to a much larger size [7,9].

Histologically, benign PTs shows an increase in the stromal cellular component, mild-moderate stromal atypia, and mitosis <5/10 high-power fields (HPFs) with a regular tumor border with no stromal overgrowth. Borderline PTs histologically show a higher percentage of stromal cellular proliferation, with more stromal atypia, and higher mitosis of 5-9/10 HPFs with infiltrating borders but lack stromal overgrowth. Malignant PTs show the highest stromal cellular proliferation with severe atypia, high mitotic count >9/10 HPFs, and diffusely infiltrative margins with stromal overgrowth [10].

Studies on breast PTs in our population are scarce, and these tumors show a wide range of morphological presentation; hence, it is important to distinguish between the subtypes of PTs so that they can be detected and managed at an early stage to improve the overall prognosis of the disease. This study aimed to evaluate various clinicopathological parameters of PTs and their association with malignancy in breast PTs.

## Materials and methods

This was a retrospective cross-sectional study conducted at the Department of Histopathology, Liaquat National Hospital, Pakistan, from January 2023 to August 2023. A total of 146 cases of PTs of the breast reported at the institute were retrospectively studied. Data on PT reported between January 2019 and December 2022 were retrieved from the institute archives. All biopsy-proven cases of PTs were included in the study. All patients enrolled in the study underwent surgical resection of the primary tumor. Clinical data, including the age and size of the tumor, were collected using the clinical referral form. The clinicopathological data of the patients included in the study were obtained from the institutional archive. Cases with missing clinicopathological data or tissue blocks were excluded from the study.

Specimens were collected by lumpectomy and simple mastectomy. After surgical resection, all specimens were sent to the laboratory in a 10% neutralized formalin-filled container for histopathological evaluation. The received specimens were evaluated grossly for tumor size, texture, color, and margins. The presence of necrosis and hemorrhage was also noted. In mastectomy specimens, skin involvement was also assessed. 

After a gross examination of the specimens, the samples were prepared for histological examination. For histological examination, paraffin-embedded tissue blocks were prepared by fixing the specimen in 10% neutralized formalin, which was then washed with water, followed by dehydration of the tissue samples with alcohol of increasing concentration. To remove alcohol from the tissue, samples were treated with xylene for three hours and then immersed in paraffin wax at 56°C. The paraffin blocks were then sliced into 3-4 μm sections. Sliced sections were transferred onto slides treated with L-lysine, sequentially treated with xylene, alcohol, and water, and stained with hematoxylin and eosin. These slides were examined by a senior histopathologist at the institute. Histological features, such as stromal overgrowth, stromal atypia, mitosis, necrosis, and heterologous elements, were assessed to determine the tumor category. Based on these histological features, the type of PT was determined. 

For most tumors, immunohistochemistry was not required. However, in a few cases of malignant PTs, in which the differential was metaplastic carcinoma, immunostaining with pan-cytokeratin was performed to exclude metaplastic carcinoma, and cluster of differentiation 34 (CD34) was performed to support the diagnosis of malignant PT. 

Data analysis

Data analysis was performed using the Statistical Package for Social Science (SPSS, version 26.0, released 2019; IBM Inc., Armonk, NY, USA). The mean and standard deviation were calculated for age, tumor size, and number of mitoses, and a one-way analysis of variance (ANOVA) was performed to determine statistical significance. Moreover, frequencies and percentages were calculated for all other clinicopathological variables. Chi-square and Fisher’s exact tests were applied to determine the association between different stages of PT and various clinicopathological features. A p-value of 0.05 was considered significant.

## Results

A total of 146 cases of PTs were included in the study. Table 1 illustrates the clinicopathological profile of the patients enrolled in the study. The mean age at the time of diagnosis in our patients was 40.65 ± 12.17 years. The patients were divided into three age groups, and our study demonstrated that PTs were more prevalent among the younger population between the ages of 31 and 50 years, accounting for 83 (56.8%) cases, whereas 34 (23.3%) patients were diagnosed at an early age of <30 years, and 29 (19.9%) cases were of a patient diagnosed at an older age of >50 years. A large proportion of tumors (*n* = 53, 36.3%) were >10 cm in size, with a mean tumor size of 9.40 ± 6.49 cm. The mean number of mitosis was found to be 11.29 ± 9.93. Specimens were obtained using two types of procedures. In the majority, lumpectomy was performed in 96 (65.8%) cases, and in 50 (34.2%) cases, specimens were obtained from simple mastectomy. Necrosis was found to occur in 21 (14.4%) cases. Stromal growth was observed in 74 (50.7%) patients. Heterologous elements were absent in the majority of cases, and only seven (4.8%) cases demonstrated heterologous elements. In the majority (*n* = 77, 52.7%) of cases, tumor margins were focally permeative, whereas diffusely permeative and non-permeative tumor margins were observed in 39 (26.7%) and 30 (20.5%) cases, respectively. Stromal atypia was found to be moderate in the majority of cases, accounting for 79 (54.1%) cases, whereas mild and severe stromal atypia was present in 63 (43.2%) and four (2.7%) cases, respectively. PT was found to be malignant in 63 (43.2%) cases, whereas 51 (34.9%) cases fell into the category of borderline tumor, and 32 (21.9%) cases were benign.

**Table 1:** Clinicopathological features of the population under study SD: standard deviation

Clinicopathological parameters	Values
Age (years), mean ± SD	40.65 ± 12.17
Age groups	
≤30 years, n (%)	34 (23.3)
31-50 years, n (%)	83 (56.8)
>50 years, n (%)	29 (19.9)
Tumor size (cm), Mean ± SD	9.40 ± 6.49
Tumor size groups	
<5 cm, n (%)	51 (34.9)
5 to 10 cm, n (%)	42 (28.8)
>10 cm, n (%)	53 (36.3)
Number of mitosis, Mean ± SD	11.29 ± 9.93
Specimen type	
Lumpectomy, n (%)	96 (65.8)
Simple mastectomy, n (%)	50 (34.2)
Necrosis	
Present, n (%)	21 (14.4)
Absent, n (%)	125 (85.6)
Stromal overgrowth	
Present, n (%)	74 (50.7)
Absent, n (%)	72 (49.3)
Heterologous elements	
Present, n (%)	7 (4.8)
Absent, n (%)	139 (95.2)
Tumor margin	
Focally permeative, n (%)	77 (52.7)
Diffusely permeative, n (%)	39 (26.7)
Non-permeative, n (%)	30 (20.5)
Stromal atypia	
Mild, n (%)	63 (43.2)
Moderate, n (%)	79 (54.1)
Severe, n (%)	4 (2.7)
Type of phyllodes tumor	
Benign, n (%)	32 (21.9)
Borderline, n (%)	51 (34.9)
Malignant, n (%)	63 (43.2)

Table 2 illustrates the association between various clinicopathological features and PT type. Benign PTs were found to affect younger patients, with a mean age of 34.12 ± 9.75 cm as borderline, and malignant PTs were diagnosed at an older age, with a mean age of 42.05 ± 11.31 and 42.82 ± 12.94 years, respectively. Hence, this demonstrates that benign tumors affect the younger population more than borderline and malignant tumors. Similarly, benign tumors were more likely to be small in size, with a mean size of 5.93 ± 5.40 cm, whereas borderline and malignant tumors were larger, with a mean size of 9.03 ± 6.69 and 11.46 ± 6.08 cm, respectively. Malignant tumors significantly displayed a higher number of mitoses, with a mean of 18.51 ± 10.74, whereas borderline and benign tumors were significantly less likely to possess mitosis. Therefore, a significant association was observed among age, tumor size, number of mitoses, and tumor type. A statistically significant association was found between the specimen type and tumor type, i.e., the majority of malignant tumor specimens were obtained by performing simple mastectomy, whereas the majority of borderline (*n* = 38, 74.5%) and benign (*n* = 30, 93.8%) tumor types chose lumpectomy. Necrosis was found to be more likely to be associated with the malignant type, present in 17 (27%) cases, compared with borderline and benign tumors. Moreover, a statistically significant association was observed between malignant tumor type and stromal overgrowth, being present in 47 (74.6%) cases compared with borderline and benign tumor types, which are less likely to possess stromal overgrowth. The malignant tumor type displayed an association with diffusely permeative tumor margins, present in 39 (61.9%) cases, whereas borderline tumors were more likely to show focally permeative margins, present in 51 (100%) cases, and benign tumors were more likely to have non-permeative margins (*n* = 30, 93.8%). Malignant tumor types were more likely to display moderate stromal atypia in 51 (81%) cases, whereas borderline and benign tumor types were more likely to possess mild stromal atypia.

**Table 2:** Association of clinicopathological parameters with the types of phyllodes tumor SD: standard deviation *One-way analysis of variance (ANOVA) was applied; **significant as <0.05; ***chi-square test was applied; ****Fisher exact test was applied.

Clinicopathological parameters	Values			p-value
	Type of phyllodes tumor			
	Benign	Borderline	Malignant	
Age (years), mean ± SD*	34.12 ± 9.75	42.05 ± 11.31	42.82 ± 12.94	0.002**
Tumor size (cm), mean ± SD*	5.93 ± 5.40	9.03 ± 6.69	11.46 ± 6.08	<0.001**
Number of mitosis, mean ± SD*	3.13 ± 0.70	7.49 ± 4.47	18.51 ± 10.74	<0.001**
Specimen type***				<0.001**
Lumpectomy, n (%)	30 (93.8)	38 (74.5)	28 (44.4)	
Simple mastectomy, n (%)	2 (6.3)	13 (25.5)	35 (55.6)	
Necrosis****				<0.001**
Present, n (%)	0 (0)	4 (7.8)	17 (27)	
Absent, n (%)	32 (100)	47 (92.2)	46 (73)	
Stromal overgrowth***				<0.001**
Present, n (%)	6 (18.8)	21 (41.2)	47 (74.6)	
Absent, n (%)	26 (81.3)	30 (58.8)	16 (25.4)	
Heterologous elements****				0.130
Present, n (%)	0 (0)	5 (9.3)	2 (3.2)	
Absent, n (%)	32 (100)	46 (90.2)	61 (96.8)	
Tumor margin***				<0.001**
Focally permeative, n (%)	2 (6.3)	51 (100)	24 (38.1)	
Diffusely permeative, n (%)	0 (0)	0 (0)	39 (61.9)	
Non permeative, n (%)	30 (93.8)	0 (0)	0 (0)	
Stromal atypia****				<0.001**
Mild, n (%)	28 (87.5)	27 (52.9)	8 (12.7)	
Moderate, n (%)	4 (12.5)	24 (47.1)	51 (81)	
Severe, n (%)	0 (0)	0 (0)	4 (6.3)	

**Figure 1:**
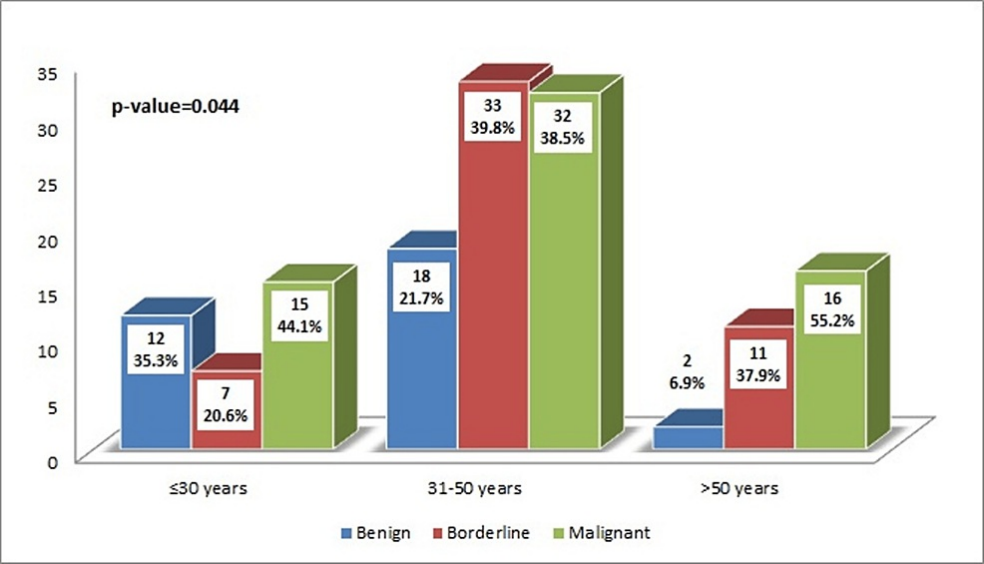
Association of phyllodes tumor type with age groups

Figure 1 demonstrates a significant association between PT category and age. The graph shows that the proportion of malignant PT is higher in the older population above 50 years (*n* = 16, 55.2%, p = 0.044) than in the younger age group. Moreover, the proportion of patients with borderline and benign PT was higher in the younger age group.

**Figure 2:**
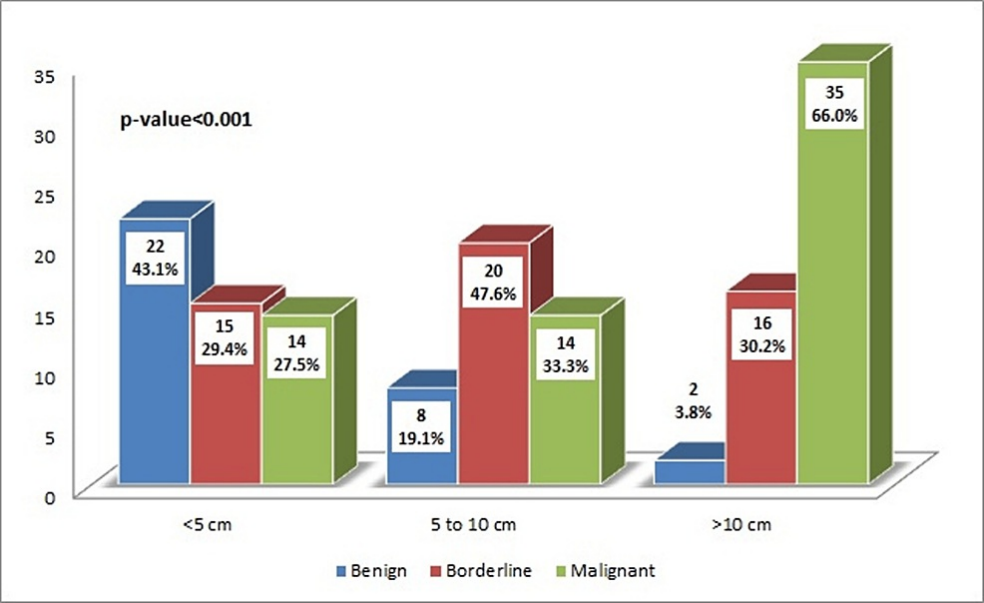
Association of phyllodes tumor type with tumor size groups

Figure 2 demonstrates a significant association between PT category and size. The graphs show that the proportion of malignant PT is higher in tumors >10 cm (*n* = 35, 66%, p < 0.01) compared with tumors of smaller size, whereas borderline and benign tumors were found to be in higher proportion in the smaller size group.

## Discussion

This study was conducted to evaluate the clinicopathological parameters for predicting malignancy in patients with PTs. We concluded that morphological features, such as necrosis, stromal atypia, stromal overgrowth, and diffusely permeative margins, were significantly more likely to be associated with malignant PTs than with borderline and benign PTs. Moreover, we found a correlation among age, size, and tumor subtype, i.e., malignant PTs were significantly more prevalent in older age groups and were larger than benign and borderline PTs, which were found to be more common in younger age groups and were of smaller size. We found that PTs commonly occurred in women in the fourth-fifth decades of life, which is consistent with previous studies conducted on PT [9].

Similar to our study, Verma et al. [11] conducted a study on PTs and found that borderline and malignant PT tumors were more commonly present in older patients than benign PTs. Karim et al. [12] analyzed the clinicopathological features of PTs in 106 cases and found that borderline and malignant PTs were more likely to be diagnosed in the older population than benign PTs. Several other studies conducted previously also supported our finding and found a correlation between age and tumor subtype and found that larger tumor size was associated with malignant PTS [13]. By contrast, previous studies have negated the association between age and tumor grade [14]. The correlation between age and tumor grade was explained by a theory that suggests that all benign PTs appear in the same age group and those that are not detected until advanced age undergo malignant transformation. Other studies suggest that benign and malignant tumors occur at different stages of life and have different biological properties [15].

The relationship between tumor size and tumor grade is still controversial. We found a significant association between tumor size and tumor subtype, i.e., we found that malignant PTs were of larger size compared with the other two subtypes. Similar to our study, few studies have previously corroborated our findings and noted an association between tumor size and PT subtype [12,15,16]. By contrast, few other studies have failed to find a relationship between tumor size and subtype [14,17].

Several studies have previously proposed that PT size is an important tool for predicting distant metastasis [9,18]. Efared et al. [19] conducted a study and analyzed clinicopathological features in 106 patients with PT. Similar to our study, they found that tumors of larger size and tumors diagnosed at an advanced age were more likely to be malignant; moreover, they concluded that histological features, such as high mitotic count, cellular atypia, and stromal cellularity, increased with tumor grade; similarly, the presence of necrosis was associated with malignant PT. Pathological features, including mitotic count, stromal overgrowth, stromal atypia, infiltrating margins, and cellular pleomorphism, are important prognostic tools and predictors of recurrence and metastasis [20].

Limitations of the study

Our study has a few limitations. First, our study included data from a single institute and had a limited sample size. Moreover, the risk factors of the tumor were not evaluated, and immunohistochemical and molecular studies of the tumors were not conducted. Because this was a retrospective study, follow-up of the disease was not performed to determine the disease-free survival rate. Therefore, we propose that a more prospective multicenter study on PTs be conducted to better understand the three subtypes of PT so that these tumors can be detected at an early stage and managed early for a better prognosis.

## Conclusions

We found that malignant PTs were more prevalent in our setup, compared with benign and borderline PTs. Moreover, a significant association was found among size, age, and tumor type, i.e., malignant PTs were more likely to be of larger size and to commonly occur at older ages compared with the benign and borderline PT subtypes. Therefore, we recommend that along with histological features, such as atypia, mitosis, and tumor borders, age and tumor size should also be considered for the risk stratification of PTs of the breast.
